# Molecular epidemiology of hepatitis B and hepatitis delta viruses circulating in the Western Amazon region, North Brazil

**DOI:** 10.1186/1471-2334-14-94

**Published:** 2014-02-21

**Authors:** Myuki Alfaia Esashika Crispim, Nelson Abrahim Fraiji, Sonia Cordeiro Campello, Nicolaus Albert Schriefer, Mariane Martins Araújo Stefani, Dagmar Kiesslich

**Affiliations:** 1 Hematology and Hemotherapy Foundation from Amazonas”/HEMOAM Research Laboratory, Amazonas Blood Center, Manaus, Brazil; 2 Federal University of Bahia, Salvador, Brazil; 3 Federal University of Goias, Tropical Pathology and Public Health Institute, Goiânia, Brazil

**Keywords:** Hepatitis delta virus, Hepatitis B virus, Genotypes, Molecular epidemiology, Western Amazon, Brazil

## Abstract

**Background:**

Hepatitis B virus (HBV) and hepatitis D virus (HDV) represent important public health problems in the Western Amazon region with reported cases of fulminant hepatitis. This cross sectional study describes HBV and HDV genotypes circulating in the Brazilian Amazon region.

**Methods:**

HBsAg positive individuals (n = 224) were recruited in Manaus/Amazonas State (130 blood donors from the Hematology and Hemotherapy Foundation from Amazonas/HEMOAM; 60 subjects from outpatient clinic) and in Eirunepe city (n = 34) from 2003–2009. Most participants (n = 153) lived in Manaus, 63 were from 20 remote isolated municipalities, 8 lived outside Amazonas State. Genotyping was based on PCR products: HBV genotype A-F specific primers, restricted length polymorphism for HDV. HDV isolates were directly sequenced (delta antigen 405 nucleotide fragment) and phylogenetic analysis performed (MEGA; neighbor-joining, Kimura’s two parameter).

**Results:**

Most participants were young adult males and HBV mono-infection predominated (70.5%, 158/224). Among blood donors, outpatient subjects and individuals from Eirunepe, HBV/A prevailed followed by HBV/D and F (p > 0.05). HBV/A was more frequent in blood donors (*p* < 0.05). HBV-HDV coinfection rate was 8.5% in blood donors (11/130), 65.0% (39/60) in outpatient subjects and 47.0% (16/34) in individuals from Eirunepe. Compared to blood donors, coinfection was higher in outpatient subjects (65.0% versus 8.5%; RR = 5.0; CI 3.4-7.9; p < 0.0001) and in subjects from Eirunepe (47.0% versus 8.5%; RR = 5.5; CI 3.0-9.9; p < 0.0001). HBV-HDV coinfection rates were higher in patients from highly endemic remote cities. Only HDV genotype 3 was detected, HBV/F-HDV/3 predominated (20/38; 52.7%), followed by HBV/A-HDV/3 (31.6%; 12/38) and HBV/D-HDV/3 (15.8%; 6/38).

**Conclusions:**

The description of HBV and HDV genotypes circulating in the western Amazon can contribute to a better understanding of their relevance on the regional epidemics. These infections are highly endemic in the Amazon where their control is challenged by its vast territorial dimension with small, hard-to-reach municipalities dispersed into the jungle and populated by diverse ethnic groups.

## Background

Worldwide more than 350 million subjects have chronic infection with hepatitis B virus (HBV) and around 15–20 million people are coinfected with hepatitis D virus (HDV) [1]. HDV is a defective RNA virus that requires the hepatitis B surface antigen (HBsAg) to become infectious [2]. HBV and HDV share similar transmission routes, primarily parenteral and sexual routes, but in areas of high endemicity as the Amazon basin, vertical and childhood horizontal transmission of HBV also occurs [3,4]. Several reports have shown that chronic HDV-HBV coinfection is associated with more severe liver disease than chronic HBV monoinfection [5,6]. HDV can be acquired by coinfection with HBV or by superinfection of HBV carrier and the clinical outcome of HDV depends on the modality of infection. Coinfection leads mostly to clearance of HBV-HDV in 95% of patients, whereas superinfection results in chronic infection in over 90% of the patients [7]. HDV is a highly pathogenic virus but the range of clinical manifestations vary from mild disease to fulminant hepatitis, as well as a rapidly progressive form of chronic viral hepatitis [8].

Delta hepatitis was endemic in southern Europe where between 1980–1990 the prevalence of anti-HDV antibodies among HBsAg-positive individuals was higher than 20%. Following the implementation of HBV vaccination programs in the 1990s a significant decline in incidence was reported [9]. However, incidence is increasing in northern and central Europe because of immigration from highly endemic areas and intravenous drug use. In the Mediterranean region, in Africa, and in the Middle East, high rates of HBsAg carriers can be coinfected with HDV whereas in the USA, HDV remains rare and restricted to high-risk groups such as intravenous drug users and hemophiliacs [10,11]. Currently eight different HDV genotypes have been reported and HDV genotype 3 is characteristic of the Amazon region in South America [12-14].

The western Amazon basin (including Brazil, Peru, Ecuador, Venezuela and Colombia) presents one of the highest rates of HBV infection in the world [15-17]. HBV is a DNA virus of high genetic variability comprising eight well-recognized genotypes, A-H and two new proposed genotypes, I and J that show distinct geographic distribution [15,18,19]. HBV genotypes together with viral load and specific viral mutations play a role in disease progression and in particular, HBV genotype can be predictive of clinical outcomes and response to interferon treatment [20]. HBV genotype A prevails in Brazil followed by genotypes D and F, the later one, prevalent in indigenous communities [21,22].

The Brazilian Amazon mainly the Juruá, the Solimões, and the Purus river basins in the State of Amazonas are considered highly endemic regions for HBV and HDV where they represent important public health issues with severe cases of acute and chronic HDV hepatitis [23-26]. Molecular epidemiology studies in this geographic region of continental dimension, including remote and hard-to-reach villages dispersed into the jungle, diverse population composed by different ethnic groups and low demographic density can help delineate improved control measures for HBV-HDV coinfection. In this context, this study describes HBV and HDV genotypes circulating in the Western Amazon region in Brazil and these data contribute to compose the molecular epidemiology map of these viruses indicating their pattern of endemicity in this highly endemic area.

## Methods

### Study area and subjects

This cross sectional study included individuals with confirmed diagnosis of HBV infection, most of them living in the western Amazon. Individuals were recruited in two cities (Manaus and Eirunepe) located in the Amazonas State, North Brazil from 2003–2009.

In Manaus city, two different groups of HBsAg positive participants were included and evaluated separately: A. Blood donors, asymptomatic subjects recruited at the main public health reference blood bank in the north Brazilian region “Hematology and Hemotherapy Foundation from Amazonas”/HEMOAM (n = 130); B. Symptomatic subjects recruited at an outpatient clinic (“Araujo Lima Hepatitis Ambulatory Hospital, Federal University of Amazonas”; n = 60). Manaus (the capital of Amazonas State) is the largest city in the Amazon region and is located in the center of the Brazilian Amazon rainforest (great metropolitan area with around 2.2 million inhabitants, situated by the Negro River a few miles before it meets the Solimões River to form the Amazon River). Manaus was historically populated by several local indigenous tribes (mainly “Manaós”) and by Portuguese and Spanish immigrants (16th century).

In Eirunepe city, HBV infected subjects were identified after a serological screening for HBsAg positivity among 720 individuals performed in 2009 (34/720; 4.72% prevalence of HBsAg positivity, Kiesslich D unpublished data). Eirunepe city (30665 inhabitants) is located 1160 kilometers away from Manaus, in the southwest part of Amazonas State by the Juruá Valley and is considered a highly endemic region for HBV and HDV infections [27,28]. Eirunepe represents a rather isolated municipality that can be reached only by the Juruá River and by airplane. It was populated during the Rubber Cycle (19th century) resulting from the miscegenation of indigenous people (Kulinaã tribe), Brazilians from the northeast region, Turkish, Portuguese and Spanish immigrants. No indigenous participant was included in any of the study groups.

### Participants

Individuals from both genders, ≥18 years old, with positive HBsAg serology (Murex, Abbot, USA) were included. Serological marker for HDV infection was determined by anti-HD IgG ELISA kit (DiaSorin, Saluggia, Italy). Anti HBV-HDV coinfection was defined by HBsAg positivity and anti-HD positivity. Exclusion criteria were: coinfection with hepatitis C virus (MUREX/Abbot, EUA), or human T cell lymphotropic virus (MUREX/Abbot, EUA), or human immunodeficiency virus (MUREX/Abbot, EUA); patients under interferon or antiviral medication, chronic alcohol consumption and illegal drug use.

### HBV genotyping

DNA was extracted from serum using silica-membrane-based nucleic acid purification kit (QIAmp DNA, Qiagen, Germany) and amplified by nested PCR. The first PCR round included primers for the complete HBV surface and in the second round, primers specific for genotypes A, B, C, D, E, F were included, as previously described [29]. HBV genotypes were identified in PCR products analyzed in 1.5% agarose gel electrophoresis.

### HDV genotyping

RNA was extracted from serum using silica-membrane-based nucleic acid purification kit (QIAmp viral RNA, Qiagen, Germany), RNA was reverse transcribed (Superscript TM One step RT PCR Platinum Taq; Invitrogen, Germany) and specific primers for nucleotide (nt) 883–906 and 1288–1265 positions of HDV genome were used [30]. For restriction fragment length polymorphism (RFLP) the amplification products were detected in 1.5% agarose gel and digested with restriction enzyme *SmaI* (5 U) (Life Technologies). Digested products were analyzed by electrophoresis through 6% acrylamide gels according to the size of the fragments: genotype 1 (227-178 bp), genotype 2 (no digestion), genotype 3 (298-107 bp) [30].

### HDV sequencing and Phylogenetic Analysis

For a subset of HBV-HDV coinfected patients, HDV isolates were submitted to direct sequencing of the partial delta antigen genomic region (405 nucleotide fragment within nucleotide positions 883–1288) using internal primers (Big Dye terminator DNA sequencing kit, Applied Biosystems, Foster City, CA) [30]. The sequences were edited manually (BioEdit V.5.0.9) and alignment, phylogenetic and molecular analyses were performed using MEGA version 4 with neighbor-joining method under Kimura’s two parameter. A bootstrap test and reconstruction was done 1000 times to confirm the reliability of the phylogenetic tree. HDV sequences from this study were deposited at the GenBank under accession numbers: KF278974 to KF278994.

### Statistical analyses

Proportions of genders, different HBV genotypes and HDV infections among groups of participants were compared by chi square and Fisher’s exact test. Comparisons of ages among groups employed ANOVA and Student’s t test. Results yielding *p* < 0.05 were considered statistically significant.

### Ethics statement

This study was approved by Institutional Review Boards (“Comite de Etica em Pesquisa da Universidade Federal do Amazonas/UFAM” and “Fundação de Hematologia do Estado de Amazonas/HEMOAM”) and all participants provided signed informed consents.

## Results

### Main features of participants

This molecular study included a total of 224 HBsAg positive individuals from the Brazilian western Amazon that were analyzed according to the recruitment site: A. Blood donors (n = 130); B. Outpatient subjects (n = 60); C. Subjects from Eirunepe (n = 34). In this study group (Table 1) males predominated among blood donors and outpatient subjects while females prevailed among participants from Eirunepe (p < 0.0001). Outpatient subjects were older than blood donors and than subjects from Eirunepe (p < 0.0007). The majority of patients (153/224; 68.30%) lived in Manaus while 63 individuals lived in 20 different remote municipalities located in the interior of the Amazonas State. Four individuals were from Lábrea, a highly endemic region for both infections. Additionally, 8 patients lived outside Amazonas State: three were from Boa Vista city capital, of Roraima State; one was from Tarauacá city, one from Sena Madureira, Acre State; two participants came from Itaituba and Tucurui, gold mining cities in Para State and one participant was from Patu city, Rio Grande do Norte State, northeast Brazil.

**Table 1:** Main demographic features, HBV genotypes in Western Amazon, Brazil

**Study group**	**Manaus**		**Eirunepe**	**Total**
	**Blood donors**	**Outpatients**		
Number (n)	130	60	34	224
Male: female#	98:32	38:22	12:22	-
Age# media, range	35 (18–60)	40 (19–66)	33 (18–50)	-
Genotyped HBV samples (n)	110	52	29	191
% HBV A (n/total)*	61.8 (68/110)	44.2 (23/52)	48.3 (14/29)	105
% HBV D (n/total)*	15.4 (17/110)	21.1 (11/52)	17.2 (5/29)	33
% HBV F (n/total)*	22.8 (25/110)	34.6 (18/52)	34.5 (10/29)	53

*p > 0.05 for Chi square comparisons of HBV genotypes among Manaus blood donors, Manaus outpatients and Eirunepe individuals; **p < 0.05 for Fisher’s exact test comparison of HBV A frequencies between Manaus blood donors and outpatients; #p < 0.001 for ANOVA comparisons of ages and Chi square comparison of male:female ratios among Manaus blood donors, Manaus outpatients and Eirunepe individuals.

### HBV infection and HBV genotypes

Overall among 224 HBsAg positive cases included in this study, 85.26% (191/224) were genotyped for HBV: 84.61% (110/130) of blood donors, 86.66% (52/60) of individuals from outpatient clinic in Manaus and 85.29% (29/34) of participants from Eirunepe.

HBV genotypes A, D and F were detected in all three groups: blood donors, subjects recruited in Manaus and in Eirunepe. The frequency of HBV genotypes identified among outpatient subjects and among participants from Eirunepe was similar (p > 0.05); therefore comparisons were performed between blood donors versus subjects from Manaus and versus subjects from Eirunepe. In these analyses, the only significant difference in HBV genotype distribution was the higher frequency of HBV/A among blood donors compared to outpatient subjects (p <0.05).

### HBV-HDV coinfection and genotypes

In this study, the majority of participants (70.5%, 158/224) had HBV mono-infection whereas around one third were HBV-HDV coinfected (Table 2). Compared to blood donors, HBV-HDV coinfection was more frequent among subjects from outpatient clinic (65.0% versus 8.5%; RR = 5.0; CI 3.4-7.9; p < 0.0001) and among participants from Eirunepe (47.0% versus 8.5%; RR = 5.5; CI 3.0-9.9; p < 0.0001).

**Table 2:** HDV-HBV co-infection and genotypes in the Western Amazon, Brazil

**Study group**	**Manaus**		**Eirunepe**	**Total**
	**Blood donors**	**Outpatients**		
Participants (n)	130	60	34	224
HBV HDV coinfection %	8.5 (11/130)	65.0 (39/60)	47.0 (16/34)	66
Genotyped HBV samples (n)	8/11	33/39	11/16	52/66*
HBV/A HDV/3	1	9	2	12
HBV/D HDV/3	3	3	-	6
HBV/F HDV/3	2	13	5	20

*Among 66 HBV-HDV co-infected individuals, 52 had HBV genotyped and 38 had both HBV and HDV genotyped.

Among 66 HBV-HDV coinfected individuals, 52 had HBV genotyped and 38 had both HBV and HDV genotyped (Table 2). HDV genotype 3 was identified in all coinfected patients recruited in the western Amazon region. HBV/F-HDV/3 predominated (20/38; 52.7%), followed by HBV/A-HDV/3 (31.6%; 12/38) and HBV/D-HDV/3 (15.8%; 6/38). The frequency of HDV/3 coinfection among blood donors was lower (RR = 0.53; CI 0.24-1.11; p = 0.054). HDV/3 coinfection was not significantly associated with HBV genotypes A or F. The relative risk of HDV/3 coinfection among outpatient subjects harboring HBV/D was borderline (RR = 0.51; CI 0.22-1.5; p = 0.07).

### HDV phylogenetic analysis

**Figure 1:**
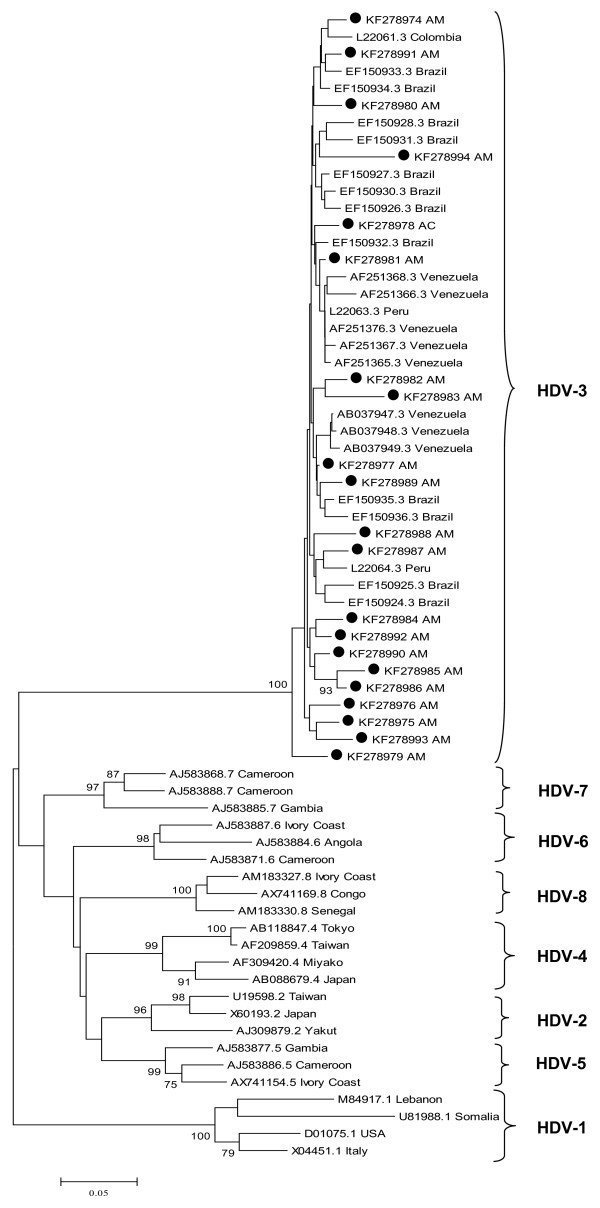
**Phylogenetic analysis of 21 HDV genotype 3 isolates from the western Amazon.** A 405 nt fragment of delta antigen (nucleotide positions 883–1288) was directly sequenced. Sequences from the western Amazon identified in this study are highlighted (●). Corresponding 405 nucleotide sub-genomic fragment of reference sequences were used in this analysis. For the reference sequences used, genotypes, origin and GenBank accession numbers of are indicated.

The phylogenetic analysis of 21 isolates showed that HDV genotype 3 sequences circulating in the western Amazon in Brazil are intermixed with sequences from other western Amazon countries as Venezuela, Colombia and Peru (Figure 1). Four HDV/3 sequences of patients coming from Lábrea region were intermixed with other genotype 3 sequences from Venezuela, Peru and other Brazilian sequences (GenBank accession numbers KF278983, KF278984, KF278988 and KF278993).

In Eirunepe, HDV genotype 3 sequences (KF278985 and KF278996) with high bootstrap were identified in individuals that did not have any clear epidemiological link, indicating a small local transmission cluster in the Juruá Valley. HDV/3 sequences from individuals living in several remote, isolated cities located in Purús, Negro and Amazonas basins indicate that this genotype is widely dispersed throughout the Amazonas State.

## Discussion

**Figure 2:**
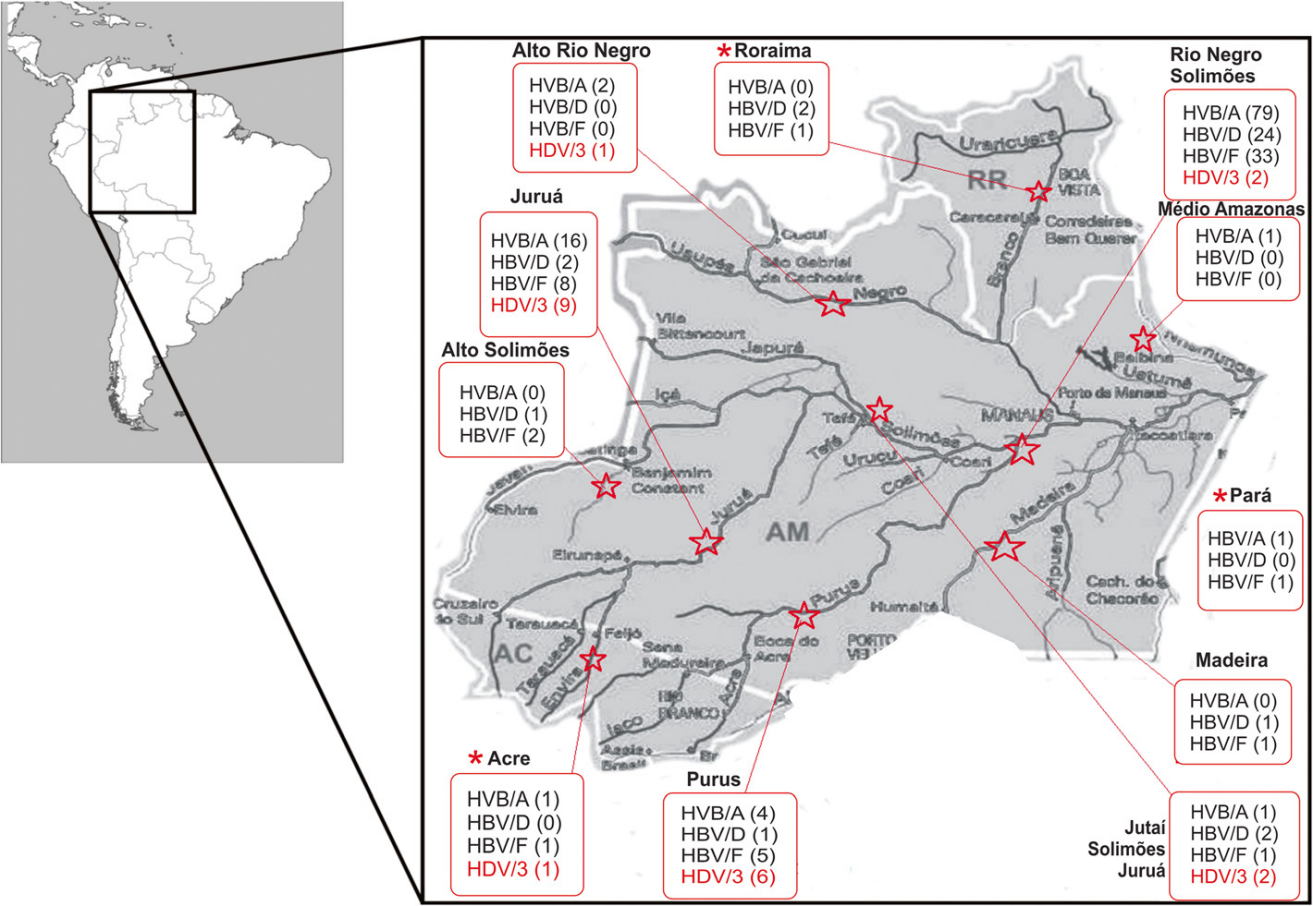
**South America map highlighting the Brazilian western Amazon region and HBV and HDV genotypes identified in Amazonas, Acre and Roraima States according to the origin of the participants, indicated by a red star.** HDV genotype 3 refers to the 21 isolates sequenced and shown in Figure 1. HBV genotypes are depicted in black and HDV genotype in red. Genotypes are shown according to the origin of participants within subregions of Amazonas State (“Alto Solimões, Jutaí/Solimões/Juruá, Purús, Juruá, Madeira, Alto Rio Negro, Rio Negro/Solimões, Médio Amazonas”). The major hydrographic basins and from the western Amazon are shown including the Negro River/Manaus, Juruá River/Eirunepe and Purús River/ Lábrea. (*) indicates genotypes in other Brazilian states in the north region: Pará, Roraima and Acre.

This molecular epidemiology study describes HBV and HDV genotypes circulating in the Brazilian western Amazon including Manaus city, the capital and largest metropolitan area of Amazonas State and several small, remote municipalities dispersed into the Amazon jungle. Blood donors from Manaus, outpatient subjects from several interior cities and subjects from Eirunepe city, located by the Juruá Valley, a highly endemic area were investigated. In this study, HBV monoinfection with genotype A predominated and genotypes D and F were also detected. HDV genotype 3 was the only genotype identified. HBV genotypes A, D and F and HDV/3 cases identified in subjects from the western Amazon depicted in Figure 2 confirm the wide distribution of these variants within this highly endemic area. In particular, HDV/3, which seems to be unique to the Amazon basin, is indeed disseminated throughout the area. Since HBV genotypes are known to impact clinical outcomes, surveillance studies remain important to identify prevailing genotypes and the possible introduction of new ones.

Brazil and Colombia were the first Latin American countries to introduce universal vaccination against HBV. In the western Amazon region, a widespread vaccination program was implemented in 1999, however, HBV and HDV infections currently remain important public health problems given the high burden reported by public health services. Additionally, the effect of HBV vaccination on mortality is not clear [31]. Even under universal HBV vaccination programs, surveillance in western Amazon should be continued to help delineate improved measures to control these infections and molecular epidemiology studies are important to define the regional pattern of endemicity. The importance of surveillance studies can be highlighted by the recent reports of high rates of HBV and HDV coinfection in developed countries mainly due to the immigration of patients from highly endemic regions and to the increased intravenous drug use [32-34]. The Amazon region attracts a significant number of migrants and tourists from all over the world and should be monitored regarding the possible introduction of different HBV and HDV genotypes that circulate in other countries, as well as their potential impact on clinical outcomes of coinfection regionally.

In this study, HBV genotypes A, D and F were identified and genotype A predominated among blood donors recruited in Manaus city. Our data corroborate previous reports showing that HBV genotype A prevails in Brazil including the northern region, followed by genotypes D and F [21,35-40]. In general, these studies have shown that about half of HBV circulating in Brazil is genotype A, probably introduced from Africa by slave trade, and in the north region, European and Lebanese immigrants have also played a role in genotype A introduction. Implications of genotype A in HBV therapy and disease progression have been well reported. Patients infected with HBV genotypes A and B have better responses to interferon-based therapy than patients infected with genotypes C and D. The predominance of HBV genotype A in the Brazilian western Amazon region, especially in Manaus, indicates that the majority of these individuals may benefit from interferon therapy.

Some molecular epidemiology studies from the Brazilian Amazon have also shown the circulation of HBV genotype F [38-40]. In the Amazon, HBV/F has been described in isolated tribes that did not have contact with non-indigenous population, whereas among tribes that had contact with non-indigenous populations, genotypes A and D have been introduced [22,41]. In our study, no indigenous participant was included and genotype F predominated among subjects recruited in an outpatient clinic that came from interior cities indicating that genotype F circulates beyond indigenous populations.

HDV/3 is considered the most divergent genotype and previous studies indicate that it is common in the Amazon Basin although genotype 1 has also been described in this region [42]. Results from the current study indicate wide dissemination and predominance of HDV genotype 3 in coinfected individuals from the western Amazon. As expected, the phylogenetic analyses showed that the HDV genotype 3 sequences from this study were intermixed with sequences from other western Amazon countries such as Colombia, Peru and Venezuela. Four HBV-HDV coinfected patients came from the highly endemic Lábrea region where a severe hepatitis outbreak caused by HDV, known as Lábrea hepatitis or black fever was reported [23]. In the current study, most coinfected patients that were genotyped harbored HBV/F-HDV/3 combination as previously reported [14], however, HBV/A-HDV/3 and HBV/D-HDV/3 coinfections were also detected. In Europe, most HBV-HDV coinfected patients harbor HBV genotype D but infection with genotype A can also occur [43]. Our study corroborates the diversity of HBV genotypes in HDV coinfection.

One interesting molecular finding was that the majority of HBV/F-HDV/3 cases from the current study was detected among subjects recruited in an outpatient clinic in Manaus. The severity of HDV infection seems to be influenced by the HDV genotypes and HDV/3 has been associated with higher pathogenicity, generally fulminant hepatitis [12,44]. In South America HBV/F-HDV/3 co-infection has been associated with increased severity of the disease. Although out of the scope of the present study, the identification of higher frequency of HBV/F-HDV/3 genotypes among symptomatic patients with liver disease seeking medical care in an outpatient clinic, confirms the poorer clinical outcomes determined by these genotypes. A previous study from our group in the same geographic region, suggested a role for HBV genotype in disease progression in which more severe liver inflammation was seen in HDV/3-HBV/F coinfected patients compared to patients mono infected with HBV/F [45]. The combination of HBV/F-HDV/3 is highly, if not invariably pathogenic, however in the current study HBV/F-HDV/3 coinfection was also detected in a low proportion of healthy blood donors. This finding indicates that this association is not "per se" pathogenic, although for unexplained reasons, the risk of liver disease in patients with this viral combination is indeed increased.

In this study, the frequency of HDV coinfection was lower in blood donors from Manaus and the higher rate of coinfection detected in Eirunepe city, probably reflects the high endemicity for these viruses already described in this region located by the Juruá Valley. PCR amplification rates and identification of HBV genotypes in this study was around 85% and around 79% for HDV. This amplification rate for HBV can be considered high, since HDV coinfection spontaneously suppresses HBV, limiting amplification rates and analysis of HBV genotypes [45,46]. Standardization of “in house”-HDV-RNA assays is important to allow comparisons of results among studies. The HDV genotype 3 sequences generated in this study and deposited in a public gene bank contribute to the somewhat limited number of HDV sequences available from this endemic region. Although a significant number of individuals participated in this study, we acknowledge that our sample size may not be representative of the vast geographic territory of the western Amazon. However, molecular studies from this region need to take into account limitations and logistic difficulties to reach and screen inhabitants of remote, small municipalities dispersed into the jungle, such as the screening performed in Eirunepe city.

## Conclusion

The description of HBV and HDV genotypes circulating in the western Amazon is important to reveal both their pattern of endemicity and their relevance on the regional epidemics. The Amazon is a highly endemic region for these infections and their control is challenged by its vast territorial dimension, with small, hard-to-reach municipalities dispersed into the jungle, and by the diverse ethnic composition of those affected. Surveillance of HBV and HDV genotypes in the western Amazon region, together with universal HBV vaccination, better educational programs about these viruses including their modes of transmission and improvements in socioeconomic conditions in these remote areas remain the cornerstone for better control strategies.

## Abbreviations

CI: Confidence interval; DNA: Deoxyribonucleic acid; ELISA: Enzyme linked immunoassay; HBsAg: Hepatitis B surface antigen; HBV: Hepatitis B virus; HDV: Hepatitis D virus; HEMOAM: Hematology and Hemotherapy Foundation from Amazonas; nt: Nucleotide; PCR: Polymerase chain reaction; RFLP: Restriction fragment lenght polymorphism; RNA: Ribonucleic acid; RR: Relative risk.

## Competing interests

The authors declare that there are no competing interests.

## Authors’ contributions

MAEC: acquisition of data, analysis and interpretation of data, drafted the manuscript critically; NAF: conception and design of the work, revised the manuscript; SCC: contributed in the acquisition of the data; NAS: analysis and interpretation of data, revised the manuscript critically; MMAS: analysis and interpretation of data, drafted and revised the article critically; Kiesslich: conception and design of the work, coordination of field and laboratory work, revised the manuscript. All authors read and approved the final manuscript.

## Authors’ information

MAEC, NAF, SCC, DK: Hematology and Hemotherapy Foundation from Amazonas/HEMOAM Research Laboratory, Amazonas Blood Center, Manaus, Amazonas, Brazil; NAS: Federal University of Bahia, Salvador, Bahia, Brazil; MMAS: Federal University of Goias, Tropical Pathology and Public Health Institute, Goiânia, Goiás, Brazil.

## Pre-publication history

The pre-publication history for this paper can be accessed here:

http://www.biomedcentral.com/1471-2334/14/94/prepub
